# Cognitive and Neuropsychiatric Impairment in Dystonia

**DOI:** 10.1007/s11910-022-01233-3

**Published:** 2022-10-06

**Authors:** Grace A. Bailey, Eva Martin, Kathryn J. Peall

**Affiliations:** 1 Neuroscience and Mental Health Research Institute, Cardiff University, Hadyn Ellis Building, Maindy Road, Cardiff, CF24 4HQ UK; 2 School of Medicine, Cardiff University, Cardiff, UK

**Keywords:** Dystonia, Cognition, Psychiatric disorders, Non-motor symptoms

## Abstract

**Purpose of Review:**

To review recent literature evaluating psychiatric and cognitive symptoms in dystonia, the two non-motor symptom groups most frequently evaluated in dystonia research and recognised in clinical practice.

**Recent Findings:**

Recent work has embedded clinical recognition of psychiatric symptoms in dystonia, with depressive and anxiety-related symptoms routinely observed to be the most common. Less explored symptoms, such as self-harm, suicidal ideation, and substance abuse, represent newer areas of investigation, with initial work suggesting higher rates than the background population. Investigation of cognitive function has provided less consistent results, both within individual dystonia subtypes and across the spectrum of dystonias, partly reflecting the heterogeneity in approaches to assessment. However, recent work indicates impairments of higher cognitive function, e.g. social cognition, and disrupted visual and auditory sensory processing.

**Summary:**

Dystonia demonstrates psychiatric and cognitive symptom heterogeneity, with further work needed to recognise endophenotypes and improve diagnostic accuracy, symptom recognition, and management.

## Introduction

**Fig. 1:**
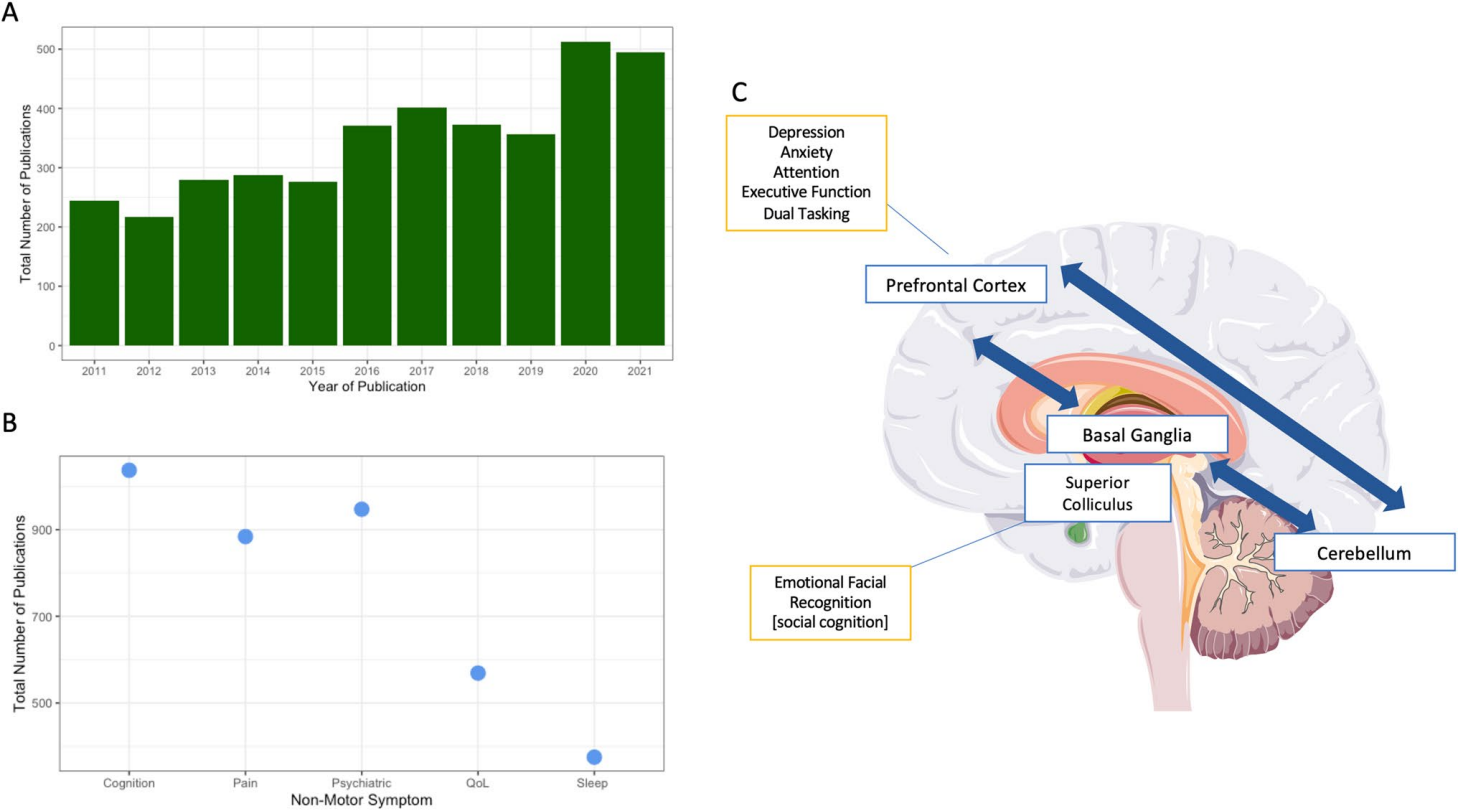
**A** Number of publications relating to the investigation of non-motor symptoms in dystonia per annum over the past 10 years (2011–2021). **B** Total number of publications examining each non-motor symptom type (psychiatric symptoms, cognition, sleep, pain, and quality of life (QoL)) over the past 10 year. **C** Schematic representation of key brain regions involved in the network-based model of dystonia and how the non-motor symptoms observed may map onto these anatomical regions

Dystonia is a movement disorder characterised by sustained or intermittent muscle contractions causing abnormal repetitive movements [1] and estimated to have a prevalence of 120/100,00 population [2]. Although predominantly defined as a motor disorder, non-motor symptoms (NMS) are increasingly recognised to contribute to the disorder phenotype, impacting social interaction, education, and health status, becoming and increased focus of dystonia research over the past 10 years (Fig. 1A). Although the spectrum of NMS is broad, those most commonly investigated include psychiatric and cognitive symptoms (Fig. 1B), both being shown to exacerbate other NMS such as pain and sleep, as well as being a primary predictors of poor health-related quality of life [3].

Multiple studies have demonstrated an excess of psychiatric symptoms across many forms of dystonia with elevated rates of depression, anxiety, obsessive–compulsive disorder (OCD), phobias, and alcohol dependence consistently identified [4–6, 7•, 8]. By contrast, investigation of cognitive deficits has revealed more varied results. Several have demonstrated multiple cognitive deficits in individuals with cranio-cervical dystonia, blepharospasm, and generalised dystonia [9–11], including attention-executive deficits [12, 13] and cognitive flexibility [14], while others have found comparable cognitive function to age-matched controls [15], or overall cognitive integrity with some deficits in specific domains, for example, executive function in patients with idiopathic and DYT1 dystonia [16]. In spite of this work, the relationship between psychiatric disorders and cognitive performance is largely poorly defined. Of the few studies that have focused on this area, findings have again varied with some identifying higher levels of anxiety impacting working memory [17] and higher depression scores associated with poorer executive function [15], while others have found no link between mood and cognition [13].

One of the central debates surrounding NMS, not only in the dystonia field, is whether these symptoms form primary or secondary components of the disorder phenotype. Some have suggested that cognitive deficits occur due to the distracting effects of motor symptoms, with evidence from *SGCE* mutation-positive myoclonus dystonia cohorts demonstrating motor symptom severity to correlate with the degree of impairment of executive function [10, 17]. However, examination of other forms of dystonia has shown cognitive function to be independent of motor deficit in those diagnosed with blepharospasm, cervical dystonia, and generalised dystonia [11, 13]. Some have observed this same mismatch between motor and neuropsychiatric symptom severity, as well as reported onset of psychiatric symptoms prior to the development of motor signs [18], and failure of psychiatric symptom improvement with the use of intramuscular botulinum toxin (BoNT) injections to improve motor symptoms [19], collectively suggesting that, at least some, psychiatric symptoms form a core component of the dystonia phenotype [3].

Here, we seek to evaluate the most recent evidence for psychiatric and cognitive symptoms in dystonia: their nature, severity, and consistency of presence, focusing predominantly on work published in the past 5 years. We will highlight areas of consistent findings, as well as those in need of greater investigation, aiming to increase awareness of the unmet clinical need and suggest directions of future study.

## Psychiatric Disorders

Over the past several decades, multiple studies have demonstrated an excess of psychiatric symptoms in dystonia compared to unaffected controls cohorts, with this being consistent whether involving focal, segmental, or generalised idiopathic forms of dystonia as well as those with an underlying genetic aetiology [20–22]. Anxiety and depression have consistently been the most common across these multiple studies and, in addition to their individual symptom burden, have also been shown to be large contributors to disability, reduced self-esteem, social interaction, and quality of life observed in those with dystonia [19].

Taking advantage of clinical-linkage approaches to examine co-morbidity at scale, two recent population-based studies have reported an excess of psychiatric symptoms across a pooled dystonia cohort, as well as when evaluating individual subtypes. Using the standardised ICD-10 classification system, these have included depressive disorders (adjusted odds ratio (aOR) = 2.00), anxiety disorders (aOR = 2.13), schizophrenia (aOR = 2.41), bipolar disorder (aOR = 1.95), and obsessive–compulsive disorder (OCD) (aOR = 1.87). This initial study also expanded its evaluation to siblings of those with dystonia, finding an increased likelihood across multiple psychiatric diagnoses compared to siblings of those without dystonia [7•]. The second of these studies exploited the opportunity to examine the temporal nature of psychiatric, relative to motor, and symptom onset. Here, psychiatric diagnoses predominantly predated motor symptom onset, particularly in the 12-month period prior to dystonia diagnosis (incident rate ratio (IRR) = 1.98), with this being most prevalent for anxiety-related disorders (IRR = 12.4), supporting findings from previous cross-sectional studies [23•, 24].

Work focused on genetically defined cohorts has predominantly involved *SGCE* mutation-positive myoclonus dystonia (DYT11) and Dopa-responsive dystonia caused by *GCH1* mutations (DYT5) in recent years. Multiple previous studies have consistently demonstrated an excess of psychiatric symptoms in both of these groups [17, 25]. However, a recent study directly compared both genetic subtypes with AOIFCD and unaffected control groups. Here, psychiatric co-morbidity in those with myoclonus dystonia differed from the other groups with a specific excess of obsessive–compulsive symptoms and psychosis, the former well documented, but the latter only previously having been reported in smaller case series [4, 26, 27]. Although mechanistic understanding remains to be determined, larger scale genetic analysis has supported susceptibility to psychiatric symptoms in genetically determined forms of dystonia, where dystonia-associated genes were enriched in nigral dopaminergic neurons and striatal medium spiny neurons, and associated with co-expression of genes linked with psychiatric disorders, including depression [28••].

### Depression

A recent, large population-based study found those with idiopathic dystonia to be twice as likely to receive a diagnosis of a depressive disorder diagnosis compared to those without dystonia (OR: 1.77; 95% CI: (1.44–2.19)). This study was also the first to extend this work to examine self-harm and suicide, demonstrating an 80% increased risk of suicide attempts/deaths in those with idiopathic dystonia, with this strongly associated with the presence of co-morbid depressive symptoms [29•]. A more recent meta-analysis aimed at determining the point prevalence of depressive symptoms or depressive disorders above clinically recognised thresholds identified 60 articles (until December 2020), 54 of which were included in the meta-analysis. Here, they divided clinical diagnoses into cervical (*n* = 28) and cranial (*n* = 14) dystonia and mixed clinical forms (*n* = 14), with the pooled prevalence for depressive symptoms calculated to be 31.5%, 29.2%, and 33.6%, respectively. Major depressive disorder was identified as the predominant symptom type in cervical dystonia and dysthymia in cranial forms. Interestingly, studies using standardised rating scales, with these including, for example, the Hamilton Depression Rating Scale and Beck Depression Inventory, identified higher levels of clinical symptom burden compared to structured interviews, with valid suggestions from the authors for need for greater standardisation of psychiatric symptom evaluation to allow for more accurate cohort comparison [30••].

Increasing numbers of imagining studies have also lent support to the existence of shared biological mechanisms underlying depression and dystonia. A voxel-based morphometry (VBM) study examining grey matter in those with Meige syndrome finding reduced grey matter volume in multiple regions including the hippocampus in those with comorbid depression compared to those without depression [31]. Abnormalities in serotonin neurotransmission were also observed in a small cohort of those diagnosed with DRD (*n* = 10), demonstrating a relationship between psychiatric symptoms and reduced hippocampal ligand binding [32]. Dopamine and its metabolites have also been implicated in dystonic symptoms with elevated levels of dopamine metabolites and lower levels of tryptophan (serotonin precursor) reported in cervical dystonia, myoclonus dystonia, and DRD compared to controls, while lower levels of levodopa have been associated with depression and more severe motor symptoms in dystonia [33]. A cross-sectional cohort study of those with DRD with and without *GCH1* mutations also found the *GCH1* mutation-positive cohort to report lower rates of self-satisfaction and higher levels of major depressive episodes prior to prescription of L-Dopa [34].

### Anxiety

Previous work consistently estimates two-thirds of dystonia cohorts to experience symptoms of anxiety [3], including both those with generalised and adult-onset isolated forms [20, 22]. Of the multiple anxiety disorder subtypes, social anxiety represents one of the more common forms, reported at rates of up to 57% in those diagnosed with adult-onset idiopathic focal cervical dystonia (AOIFCD) [8, 22], with some links to the social stigma associated with body concept and subsequent impact on quality of life [35]. This is consistent with recent findings which support higher levels of anxiety and depression in those diagnosed with AOIFCD with greater social perception [36••], while a single study of those with musician’s dystonia has postulated that higher levels of anxiety may be a coping mechanism related to perfectionistic behaviour [37].

As outlined above, population-level studies have demonstrated diagnosis of anxiety disorders prior to the motor diagnosis of dystonia, while another recent study also suggested that earlier onset motor symptoms are observed in those where mood disorders develop prior to the onset of the motor symptoms [38, 39]. Another investigation aimed at examining diagnostic delay in dystonia found that those with AOIFCD were significantly more likely to be diagnosed with depression and anxiety in the 5-year period prior to their motor diagnosis, compared to unaffected controls [40]. Interestingly, timeliness of initiation of motor symptom treatment also appears to impact depressive symptoms in this cohort with improvement in mood disorder symptoms following injectable neurotoxin treatment in those who were diagnosed within 5 years of dystonia symptom onset compared to those with motor symptoms for > 5 years prior to diagnosis [8].

### Other Psychiatric Symptom Groups

Others have examined less frequently reported psychiatric symptom profiles in dystonia. A cross-sectional study comparing the musician’s dystonia (*n* = 101) to a mixed group of other focal dystonias (*n* = 85) found that although the overall rates of psychiatric symptoms in those with the musician’s dystonia was half that of focal dystonia (27.8% vs. 5.3%), with examination of personality disorder profiles, albeit using a single questionnaire (the Neuroticism Extraversion Openness Five-Factor Inventory) found significantly higher neuroticism and openness scores in women (*p* = 0.029) and men (*p* < 0.001) with the musician’s dystonia, respectively [41]. Another examined rates of substance abuse in those with AOIFCD, finding overall rates to be 11% (*n* = 208), with over a quarter of this group taking opiate analgesia (*p* = 0.006 compared to AOIFCD with no reported substance abuse). Those with symptoms of substance abuse were also more likely to be male (*p* = 0.0007), younger (*p* = 0.031), and have a higher burden of psychiatric symptoms, including depression (*p* = 0.005) and anxiety (*p* = 0.003). However, those with reported substance abuse also reported higher overall disability scores and had higher motor symptom severity and pain scores, likely accounting, at least in part for this excess analgesic use [42].

## Cognitive Symptoms

As can been seen in Fig. 1A and B, cognitive symptoms not only are the most commonly investigated NMS alongside psychiatric disorders in dystonia, but in recent years greater emphasis has been placed on examining features of higher cognitive function. In comparison with psychiatric symptoms, results from cognitive studies have been less consistent with suggestion that greater heterogeneity is observed between types of dystonia [17, 43•, 44]. Multiple cross-sectional studies exemplify this variation with some finding no difference in questionnaire-derived measures of impulsiveness (*p* = 0.65), overall cognition (Montreal cognitive assessment, *p* = 0.14), and executive function (*p* = 0.42) between AOIFCD and controls [45], while others examining a broader clinical group of primary dystonias found significantly poorer performance across multiple measures including global cognitive function (*p* < 0.001), attention (*p* < 0.001), memory (*p* < 0.001), and conceptualisation (*p* = 0.001), compared to unaffected controls [46]. Others, even when examining multiple cognitive domains (e.g. prospective and verbal memory, executive function, subjective prospective, and retrospective memory) across multiple dystonia types (cervical dystonia (*n* = 26) and blepharospasm (*n* = 27), compared to an unaffected control cohort (*n* = 30)), have found differences only in a single domain, here being significantly poorer prospective memory across both time-based (*p* < 0.001) and recognition tasks (*p* = 0.013), indicating potential dysfunction in processing of the prefrontal cortex [47]. However, three areas have emerged as the main fields of research focus over recent years, namely, visual and auditory processing, social cognition, and dual tasking, outlined in further detail below.

### Visual and Auditory Processing

Multiple previous studies have indicated dysfunction in the processing of sensory input, with this being attributed to the loss of lateral inhibition in sensory areas of the brain [48, 49]. Chillemi and colleagues examined whether processing of spatial and temporal perception in those diagnosed with AOIFCD (*n* = 21) compared to healthy controls (*n* = 22). However, the authors subdivide the AOIFCD group clinically into much smaller individual groups: laterocollis (*n* = 9), torticollis (*n* = 9), and tremor (*n* = 3), identifying lower accuracy in the spatial processing of the laterocollis group (*p* = 0.021) and minimal reduction in temporal processing in the torticollis subgroup (*p* = 0.043), albeit with no correction for multiple testing [50].

Cross-sectional evaluation of visual attention, functional vision, and overall cognitive function in another AOIFCD cohort found no difference in overall cognitive function but significantly lower visual processing speeds (*p* < 0.001), divided attention (*p* < 0.001), and selective attention (*p* = 0.001), suggesting a reduction in functional vision and visual attention in AOIFCD [51]. Further work from another group explored visuospatial attention as a measure of higher level visuospatial processing in AOIFCD (*n* = 23, compared matched controls, *n* = 12), finding those with torticollis to have a higher level of leftward deviation, potentially suggesting an underlying lateral bias to the spatial attention deficits observed [52].

Gene-specific studies, again focused on *SGCE* mutation-positive myoclonus dystonia have also suggested disruption to normal visual sensory processing in its underlying pathogenesis. Here, an *SGCE*-positive cohort with (*n* = 13) and without (*n* = 24) deep brain stimulation (DBS) was compared to unaffected controls in evaluating temporal discrimination thresholds, movement orientation, and speed discrimination. Although once again the cohort examined was small, this study benefited from the genetic homogeneity of the *SGCE*-positive cohort with sensory mean growth rate, a measure of sensory accumulation in the processing of visual information, significantly lower in this group compared to controls across all three measures (*p* < 0.01). In those without DBS, the sensory mean growth rate in the temporal discrimination threshold was also negatively correlated with the thickness of the primary visual cortex, which itself was significantly thicker compared to unaffected controls (*p* = 0.001), collectively supporting the key role that sensory processing, likely in its multiple formats, plays in giving rise to, or propagating dystonia pathogenesis [53].

### Social Cognition

The sensory deficits outlined above, and, in particular, previous work demonstrating temporal discrimination deficits, suggest involvement of the superior colliculus in the altered network function in dystonia. The superior colliculus is also believed to be involved in the processing of emotional facial recognition, with recent research examining for potential deficits in social cognition in dystonia [54]. Social cognition is described as the ability to attribute affect and mental states to others, combining multiple cognitive processes including an ability to recognise emotion and to distinguish between the intentions of others and self. Theory of mind (ToM) is central to social cognition and is defined as an ability to understand and interpret intentions, emotions, and beliefs of others to accurately predict their behaviour and act accordingly. Deficits in social cognition could potentially indicate dysfunction in the collicular-pulvinar-amygdala pathway, which may also account for other NMS observed, including temporal discrimination, anxiety, and depression.

Research in this area has tended to focus on AOIFCD, likely due to its prevalence as well as anecdotal clinical observation that social cognitive deficits may be more evident in adult-onset focal forms of dystonia. However, a single study of Mendelian inherited dystonia (*SGCE*, myoclonus dystonia) identified lower emotional recognition scores to a SGCE-negative movement disorder cohort [55]. A cross-sectional case-controlled study of age-, sex-, education-, and IQ-matched cohorts (*n* = 46 in each group) found significantly poorer performance of the AOIFCD cohort in delayed recall (*p* < 0.001), recognition (*p* = 0.006), and basic social cognition (*p* = 0.007) where participants were asked to correctly label affect (happy, sad, fearful, or neutral) for both visual and auditory stimuli [56]. A second study compared those with AOIFCD and no depressive symptoms (*n* = 25) to unaffected controls (*n* = 26), finding the AOIFCD cohort to demonstrate significantly reduced ability in inferring both cognitive and affective status, with understanding the intentionality of others being the most prominent area of difference and unrelated to motor symptom severity [57]. Examination of wider social cognitive abilities, including social perception, ToM, empathy, and social behaviour, alongside anxiety and depression, found 21.74% of the AOIFCD (*n* = 46) cohort demonstrated impaired belief reasoning and 5/46 (10.87%) impaired empathy. Interestingly, those with more severe anxiety and depressive symptoms tended to have greater social perception abilities, suggesting some adaptation of social cognitive skills [36••]. Finally, separation of an AOIFCD cohort into those with (*n* = 35) and without (*n* = 14) tremor found significantly poorer performance in the affective(*p* = 0.009) and cognitive (*p* < 0.00001) domains of the ToM tests in the AOIFCD cohort compared to controls, however, with the presence of a tremor only demonstrating an effect in the cognitive domain (*p* = 0.03) [58].

### Dual Tasking

Assessment of dual tasking (performance of two or more tasks) has been extensively examined in other types of movement disorders with this providing an increasing focus for dystonia research, due to the increasing evidence of sensory processing and sensorimotor integration outlined above. Dual tasking is also dependent on higher cognitive processes such as executive functions required for planning and executing complex task, suggesting prominent cortical involvement, with some suggesting that impaired dual tasking suggests a reduction in contingency or reserve in those regions involved in dual tasking. Again, most studies have focused on AOIFCD, identifying impaired postural control with increasing cognitive task complexity compared to controls (*p* < 0.0001) with neither dystonia motor severity nor the presence of a tremor being significantly associated with the degree of impaired stability [59]. Further studies have extended this to evaluate the impact of dual tasking on both balance and gait, again examining those with AOIFCD (*n* = 17), compared to controls (*n* = 19), at normal walking speed, fast walking, cognitive dual task, and obstacle negotiation. Those with AOIFCD were found to have significantly slower gait speed, longer stance time, and dual task cost, compared to controls, with the changes to the gait parameters correlated with balance ability, and once again not associated with dystonia motor symptom severity [60].

## Comparison Between Distinct Forms of Dystonia

As can be seen above, most of the studies outlined have focused on cross-sectional analysis of a single form of adult-onset idiopathic dystonia or inherited form. However, several studies have suggested that not only are phenotypic subgroups likely within individual diagnoses, the relative contribution of non-motor symptoms (both primary and secondary) to the overall disorder phenotypic spectrum is likely to differ between type of dystonia [5, 43•, 61]. As such several studies have compared psychiatric and cognitive symptoms between dystonia diagnoses with one comparing AOIFCD (*n* = 25), generalised dystonia (*n* = 13), and unaffected controls (*n* = 50), with the combined dystonia groups demonstrating impairment across a single cognitive task—the trail making test—but with no between dystonia group difference. The study concluded that overall cognitive symptoms, but elevated rates of mood disorders were observed in both dystonia groups, with these independent of motor symptom severity and disability [62]. Again, evaluation of a larger (blepharospasm *n* = 60, cervical dystonia = 60) cohort, cognitively assessed using the Addenbrooke’s Cognitive Examination Revised (ACE-R), with cognitive impairment defined as a total score < 75, found significantly higher rates of cognitive impairment in the dystonia group compared to controls (*p* < 0.001), but not between dystonia subtypes [63].

Two studies have undertaken cluster analyses of AOIFCD cohorts, combining motor and non-motor symptoms, aimed at identifying phenotypic subgroups. Although neither study involved large cohorts, and were likely underpowered for this form of analysis, both identified two main clusters. The first study found psychiatric symptoms, namely, anxiety and depression, to differ between the clusters, together with other NMS such as pain and sleep disturbance [5]. The clusters in the second study instead differed on NMS severity, age, and degree of disability [61]. Interestingly, both studies found sex and motor symptom duration not to differ significantly between clusters, lending some support that at least some of these symptoms driving this subgrouping represent primary phenotypic components of dystonia.

## Impact of Treatment on Psychiatric and Cognitive Symptoms

Studies assessing the relationship between the neuropsychological and motor traits in dystonia have consistently found poor levels of association with disease duration and motor severity [5, 20]. As such, several studies have sought to evaluate the impact of treatment aimed both at motor and non-motor symptom management on both psychiatric and cognitive symptoms. Multiple studies have assessed the long-term impact of deep brain stimulation (DBS) with one finding no statistical difference between premorbid levels of anxiety and depression and those at 2-year follow-up in those diagnosed with AOIFCD [64]. By contrast, studies focused on myoclonus dystonia have found both no improvement and, in one case, deterioration of mood symptoms, in comparison with baseline, at follow-up assessment [65, 66]. Finally, a cohort implanted with constant current DBS (*n* = 10) underwent neuropsychological evaluation at 1, 6, and 12 months post-operatively with no significant difference in any measure at 1-month post-surgery; however, at 6 months, there was improvement in phonemic fluency compared to baseline (*p* < 0.05), with this preserved at 12 months [67].

Few trials have evaluated the impact of oral medical therapy, typically used in the management of mood-related symptoms, in the management of psychiatric symptoms in dystonia. Building on previous work that demonstrated reduced serotonin transporter (SERT) binding during FP-CIT-SPECT scans of those with cervical dystonia compared to unaffected controls [68], an initial randomised control trial investigating the use of escitalopram compared to placebo, alongside the patient’s routine BoNT treatment for cervical dystonia, for a period of 6 weeks unexpectantly found no improvement to either motor or psychiatric symptoms, potentially due to the limited period of follow-up evaluation [69]. A subsequent double-blind placebo-controlled trial from the same group and involving 8 participants in each arm (escitalopram and placebo) found no significant difference in the SERT, DAT, or D2/3R binding with SPECT imaging, but those who reported improved motor or psychiatric symptoms following treatment with escitalopram had a significantly higher SERT occupancy than those who reported no change (76.5% vs. 67.5% occupancy respectively, *p* = 0.04) [70].

Cognitive-behavioural treatment (CBT) options are also underutilised in dystonia, with no standardised management available. A small, randomised feasibility trial explored the use of unguided Internet-based CBT in the management of AOIFCD. This work demonstrated that this approach to delivery of CBT was feasible in an AOIFCD cohort, with 60% demonstrating high engagement with the 8-week programme and 87.5% of those who engaged would try it again if offered. Although this study was not designed, not sufficiently powered, to identify between group differences in terms of the efficacy of the treatment, the levels of anxiety and depression showed trends towards improvement at 3 months in those receiving iCBT. Individual level analysis also demonstrated higher percentage level improvements in these symptoms, with this sustained in 86% participants, making this a potentially promising area for future investigation [71, 72]. A single RCT investigated the long-term effectiveness of physical therapy in an AOIFCD cohort. In addition to demonstrating improvement in their motor symptoms, symptoms of pain, anxiety, and depression remained significantly improved 12 months after instigation of the programme, with these improvements potentially related to enhanced neuroplasticity [73], whereas a pilot study found reduced symptoms of depression but no improvement in anxiety symptoms following a relaxation programme [74].

## Conclusion

Psychiatric and cognitive symptoms remain an area of focus in dystonia research, with increasing evidence to suggest that at least a portion of the psychiatric symptoms observed form a primary component of the dystonia phenotype. Our understanding of cognitive deficits is less clear, with these more likely to involve higher cognitive function, as well as demonstrating greater variability in phenotype and severity both between and within individual subtypes of dystonia. The key to better management and therapeutic intervention is mechanistic understanding, and how cognitive, psychiatric, and motor symptoms may arise from common biological pathways or involvement of overlapping regions within the same network. Work to date suggests cortical regions, notably the prefrontal and frontal regions, and cortico-striatal circuits, may represent areas of anatomical interest, but further work is needed to refine these hypotheses (Fig. 1C). Future studies need also to develop consistency in the approach to psychiatric and cognitive symptom assessment, allowing for between cohort comparison and facilitating multisite collaborative studies, while also enabling longitudinal analysis of symptom variation. Finally, emerging evidence suggests NMS may form symptom group clusters within individual dystonia diagnoses. This warrants further investigation in larger cohorts, potentially allowing for more targeted and individualised treatment within the clinical setting.

## Declarations

### Data Access Statement

No new data was created during this review.
